# Allostatic load elevates the risk and adverse prognosis of immune-mediated inflammatory diseases: modulatory effects of lifestyle interventions and genetic susceptibility

**DOI:** 10.1016/j.jnha.2026.100792

**Published:** 2026-01-24

**Authors:** Ziling Yang, Jinming Zhang, Zhong Qu, Zhuo Zhao, Yajuan Zheng

**Affiliations:** a The First Hospital of Jilin University, Jilin University, Changchun 130021, People's Republic of China; b The Second Hospital of Jilin University, Jilin University, Changchun 130000, People's Republic of China

**Keywords:** Psychological stress, Autoimmune diseases, Lifestyle factors, Lifestyle interventions, Genetic predisposition, Cohort studies

## Abstract

**Background:**

Allostatic load (AL)—the cumulative biological cost of lifelong stress—can disrupt immune homeostasis via hypothalamic–pituitary–adrenal-axis dysregulation and persistent sympathetic activation. Immune-mediated inflammatory diseases (IMIDs) are organ-specific chronic inflammatory disorders imposing a major public-health burden, yet their causal link with AL remains unclear.

**Methods:**

In this prospective study of 186 310 UK Biobank participants, Cox proportional-hazards models quantified dose-response associations between AL and the incidence of ten IMIDs plus all-cause mortality. Interaction models evaluated the modifying effects of physical activity, ω-3 polyunsaturated fatty acids (ω-3 PUFAs) and other lifestyle variables, and assessed gene–environment interplay using polygenic risk scores (PRS).

**Results:**

Compared with the lowest AL quartile, the highest quartile showed significantly greater incidence of rheumatoid arthritis (hazard ratios (HR) = 1.52), spondyloarthritis (HR = 2.50), asthma (HR = 1.38), inflammatory bowel disease (IBD) (HR = 1.19), type 1 diabetes (T1DM) (HR = 5.16), psoriasis (HR = 1.87), autoimmune retinopathy (HR = 1.77) and composite IMIDs (HR = 1.55) (all p < 0.05). Elevated AL also predicted dose-dependent increases in all-cause mortality among patients with rheumatoid arthritis (HR = 6.59), asthma (HR = 1.87), IBD (HR = 2.00), T1DM (HR = 2.72) and composite IMIDs (HR = 2.01). Sufficient physical activity and higher ω-3 PUFA intake partially attenuated AL-related risks, whereas high PRS synergistically amplified AL effects for spondyloarthritis (attributable proportion (AP) = 7.6%), T1DM (AP = 4.7%) and psoriasis (AP = 4.9%).

**Conclusions:**

AL is causally linked to both the development and prognosis of IMIDs, with its impact jointly modifiable by lifestyle factors and genetic susceptibility. Building AL-centred psychoneuroimmunological biomarker networks may enable refined risk stratification and precision interventions for IMIDs.

## Highlights

• Allostatic load (AL) elevates immune‑mediated inflammatory disease (IMID) risk.
• Physical activity and ω‑3 intake partially offset AL‑linked immune disease risks.
• Stress‑gene synergy identified: polygenic scores amplify AL effects on IMIDs.

## 1 Introduction

Immune-mediated inflammatory diseases (IMIDs) constitute a class of clinical disorders marked by aberrant activation of the adaptive immune system and organ-specific chronic inflammation. This spectrum encompasses rheumatoid arthritis (RA), the spondyloarthritis (SpA) family, connective-tissue diseases, type 1 diabetes mellitus (T1DM), cutaneous inflammatory disorders such as psoriasis, inflammatory bowel disease (IBD), asthma, autoimmune ocular conditions such as autoimmune retinopathy (AIR), and neurological diseases including myasthenia gravis (MG). Although individual IMIDs present heterogeneous clinical phenotypes through shared immune pathways (e.g., the TNF-α/Th17 axis) or disease-specific mechanisms, they share core attributes—namely a chronic relapsing course, progressive tissue damage, and multisystem involvement [1]. Epidemiological evidence indicates that roughly 5 % of the global population lives with at least one IMID, posing a substantial, systemic medical challenge [2].

Recent advances in psychoneuroimmunology suggest that chronic stress can disrupt immune homeostasis via dysregulation of the hypothalamic–pituitary–adrenal (HPA) axis and hyperactivation of the sympathetic nervous system [3]. Pre-clinical studies employing the social-defeat mouse model confirm that exposure to psychological stressors triggers abnormal innate immune activation—manifested by elevated circulating interleukin-6 (IL-6) and expanded myeloid cell counts—and reprograms the adaptive immune system, as evidenced by enlarged memory-T-cell pools after infection [4]. The concept of allostatic load (AL) offers a quantitative framework for capturing the cumulative impact of stress. A semi-structured AL interview scale grounded in clinical psychometrics has been incorporated into the Diagnostic Criteria for Psychosomatic Research (DCPR) [5]. AL reflects the physiological cost incurred when neuro-endocrine systems are persistently or excessively mobilized to cope with environmental challenges. Spanning multiple physiological systems, AL has become a key construct for elucidating how chronic stress translates into pathophysiology [5]. Elevated AL scores have been epidemiologically linked to earlier health deterioration, increased cardiovascular risk, and greater cancer susceptibility, among other adverse outcomes [5,6].

Against this backdrop, we plan to leverage the large-scale UK Biobank cohort to investigate dose–response relations between AL and both IMID incidence and all-cause mortality, employing Cox proportional-hazards models. Multifactorial interaction models will further explore whether lifestyle interventions—namely ω-3 polyunsaturated fatty-acid (ω-3 PUFA) intake, healthy sleep patterns, and regular physical activity—can buffer AL-related immune dysregulation. Finally, polygenic risk scores (PRS) will be used to delineate the moderating influence of genetic susceptibility within gene–environment interactions.

## 2 Method

### 2.1 Study design and population

Between 2006 and 2010, the UK Biobank recruited ∼500,000 community-dwelling adults (predominantly middle-aged and older) from 22 assessment centres across the United Kingdom and established a large prospective cohort. At the baseline visit, participant information was collected through touchscreen questionnaires and structured interviews, alongside standardised physical measurements and physiological assessments; biological samples were collected and stored to support genotyping and a wide range of biochemical and metabolomic assays, creating a multidimensional resource spanning self-reported data, objective measurements, and biomarker profiling. Long-term follow-up for health outcomes is primarily achieved through linkage to routine electronic health records and national registries (e.g., hospital inpatient records, cancer registrations, death registrations, and, where available, primary care records), enabling ascertainment of disease occurrence and timing. Because linkage coverage and censoring dates can differ across data providers and UK nations, the available follow-up duration may vary between participants [7].

The present analysis uses UK Biobank data under application 84347. Ethical approval was granted by the North-West Multi-centre Research Ethics Committee (ref. 16/NW/0274), and all participants had provided written informed consent at enrolment.

**Fig. 1:**
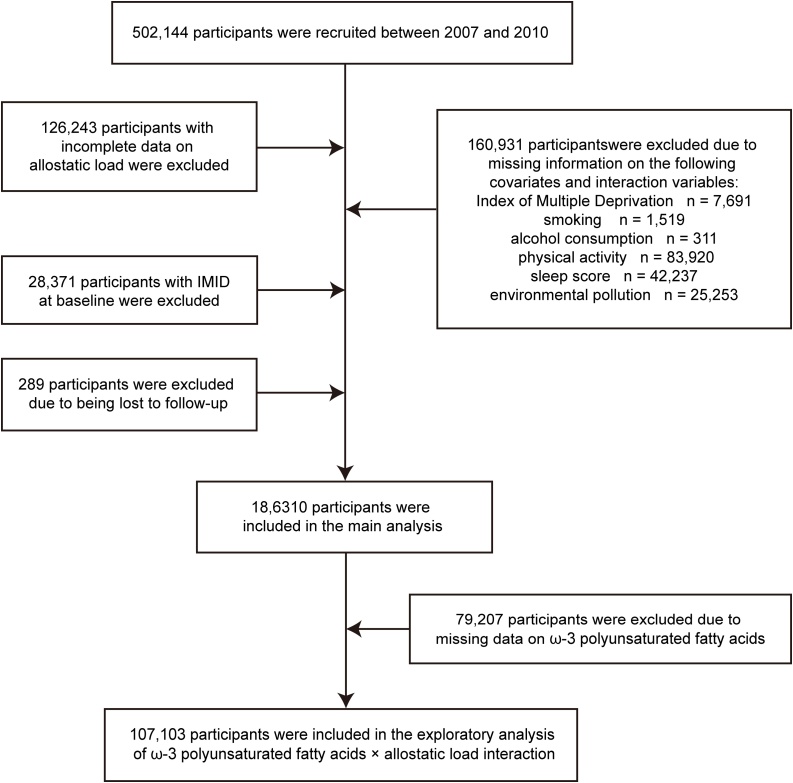
Flowchart of the study. IMID: immune-mediated inflammatory diseases.

Of the 502,144 eligible participants, we excluded individuals who (i) were lost to follow-up; (ii) lacked any biomarker required for the AL index; (iii) were missing covariate data; or (iv) had been diagnosed with any outcome disease before baseline. A detailed flow chart of participant selection is shown in Fig. 1.

### 2.2 Exposure assessment—AL

AL is a composite measure designed to quantify an individual’s long-term, cumulative physiological burden of stress across multiple biological systems. Several commonly used frameworks for constructing AL have been described in prior studies [8]. Based on the biomarkers available in the UK Biobank, we included 12 indicators spanning metabolic, cardiovascular, inflammatory/immune, and renal function domains: glycated haemoglobin (HbA1c), triglycerides, high-density lipoprotein cholesterol (HDL-C), low-density lipoprotein cholesterol (LDL-C), total cholesterol, waist-to-hip ratio, systolic blood pressure, diastolic blood pressure, pulse rate, C-reactive protein, insulin-like growth factor 1 (IGF-1), and creatinine [8] (Table S1).

AL was scored using an internal, cohort-specific quantile approach. For 10 biomarkers (all except HDL-C and IGF-1), values at or above the 75th percentile were assigned 1 point; for HDL-C and IGF-1, values at or below the 25th percentile were assigned 1 point. Points were summed across the 12 biomarkers to derive the total AL score (range: 0–12) [8]. To comprehensively characterise dose–response relationships and facilitate clinical interpretation, AL was operationalised in three ways: (1) as a continuous variable; (2) categorised into quartiles; and (3) dichotomised at the cohort median into high versus low AL (with high AL defined as above the cohort median).

### 2.3 Outcome definition—IMIDs

Following established criteria [1], we examined ten common IMIDs: RA, SpA, asthma, IBD, SLE, T1DM, MG, psoriasis, uveitis, and AIR. A diagnosis of any one of these conditions was classified as “any IMID” to evaluate the overall association with AL. The corresponding clinician-assigned ICD-10 codes are listed in Table S2.

### 2.4 Covariates and interaction terms

Covariates and potential effect modifiers were primarily derived from the UK Biobank baseline assessment and biospecimen assays. Demographic and lifestyle characteristics were obtained via touchscreen questionnaires and nurse-led interviews, while anthropometric measures and selected physiological parameters were collected through standardised on-site examinations. Blood and other biological samples were used for genotyping and a wide range of biochemical/metabolic assays. In addition, selected environmental exposure metrics were generated by spatially linking participants’ residential addresses to external environmental models and databases [9].

In the primary analyses, we adjusted for the following baseline covariates: age (continuous), sex, ethnicity, socioeconomic status, smoking, and alcohol use. Age was defined as the participant’s attained age at recruitment. Sex was determined using a combination of National Health Service (NHS) records and self-report. Ethnicity was based on self-reported ethnic background; given the relatively small proportion of non-White participants and to ensure stable estimation, we dichotomised this variable as White versus non-White (including Mixed, Asian/Asian British, Black/Black British, Chinese, and other ethnic groups). Socioeconomic status was captured using the Index of Multiple Deprivation (IMD), which ranks neighbourhood-level deprivation based on postcode-linked small-area geographies; higher scores indicate greater deprivation, and IMD was modelled as a continuous variable. Smoking status was classified as never versus former/current smoking based on self-report. Alcohol use was dichotomised using UK Biobank–recorded alcohol units and thresholds from UK dietary guidelines: participants were classified as “lower risk/within guideline” if intake did not exceed 1 drink per day for women or 2 drinks per day for men, and as “higher risk/above guideline” otherwise [10].

For effect modification analyses, we focused on whether lifestyle factors, environmental exposures, and genetic susceptibility modified the association between AL and IMIDs. Lifestyle modifiers included physical activity and a healthy sleep score. Physical activity was assessed using the International Physical Activity Questionnaire–Short Form (IPAQ-short) and categorised as meeting versus not meeting recommended activity thresholds. The healthy sleep score was constructed in accordance with prior UK Biobank research and incorporated five components: sleep duration (7–8 h/day), chronotype (morning preference), insomnia (rarely/never), no snoring, and daytime sleepiness (rarely/never). Participants meeting all five criteria were classified as having low-risk sleep behaviour; all others were classified as high-risk [11].

Environmental modifiers included residential noise exposure, green/blue space, and a composite air pollution exposure metric. Noise exposure was defined using the 24 -h average environmental noise level and, to aid interpretability, was rescaled per 10-unit increase. Green/blue space was estimated using 2005 Generalised Land Use Database (GLUD) data to derive the proportions of green land and water within a 300 m buffer around each residential address; the green-to-blue space ratio was used as the exposure metric and was similarly rescaled per 10 units. Composite air pollution exposure was derived from land-use regression (LUR) model–estimated annual mean concentrations of particulate matter with aerodynamic diameter ≤2.5 μm (PM2.5), particulate matter ≤10 μm (PM10), nitrogen dioxide (NO2), and nitrogen oxides (NOx). These pollutants were combined into an overall exposure score using regression coefficient–based weighting from multivariable Cox proportional hazards models to capture the joint effect of multiple pollutants [12,13].

At the biomarker level, we used targeted high-throughput nuclear magnetic resonance (NMR) metabolomics (Nightingale Health) to quantify non-fasting EDTA plasma omega-3 polyunsaturated fatty acids (ω-3 PUFA) and docosahexaenoic acid (DHA), and categorised these measures into tertiles [14]. At the genetic level, we constructed outcome-specific polygenic risk scores (PRS) using a clumping-and-threshold approach for linkage disequilibrium (LD) pruning and p-value filtering (1,000 kb window, r^2^>0.1, retaining genome-wide significant variants at p ≤ 5 × 10^−8^). PRS were then grouped into low (lowest quintile), intermediate (second to fourth quintiles), and high (highest quintile) genetic risk strata. UK Biobank field IDs, threshold definitions, and supporting references for all variables are provided in Table S3.

### 2.5 Statistical analysis

Continuous variables are presented as mean (standard deviation), and categorical variables as number (percentage). The primary association analyses used Cox proportional hazards regression to examine the relationship between baseline AL and incident IMIDs. Follow-up began on the date of the baseline assessment. Outcomes were ascertained through UK Biobank linkages to routine healthcare records and registries, with the event time defined as the date of first diagnosis of the corresponding IMID. Participants were followed until the earliest of: first occurrence of the outcome of interest, death, loss to follow-up, or administrative censoring at the latest available linkage date (12 September 2024). To help readers interpret the follow-up window and the accrual of events, we report the median follow-up time (interquartile range) in the Results.

After accounting for potential confounders (age, sex, ethnicity, IMD, smoking, and alcohol use), we estimated the main effect of AL using a single fully adjusted model that included all prespecified covariates simultaneously, and present results as hazard ratios (HRs) with 95% confidence intervals (CIs). Because stepwise or sequential model building is a data-driven selection process that can lead to model instability, biased estimates, and reduced reproducibility, we prespecified the confounder set a priori based on epidemiological evidence and adopted a “prespecified covariates + single fully adjusted model” strategy to improve interpretability and internal consistency [[15], [16], [17]].

We then evaluated whether the interaction variables modified the association between AL and IMIDs (and, among participants with IMIDs, all-cause mortality) on either the additive or multiplicative scale. Additive interaction was quantified using the relative excess risk due to interaction (RERI) and the attributable proportion due to interaction (AP), with 95% CIs estimated using the delta method. Multiplicative interaction was assessed by including a product term between AL and the interaction variable in the Cox model.

To assess robustness, we conducted two sensitivity analyses. First, to reduce potential reverse causation or bias from subclinical disease present but undiagnosed at baseline, we performed a lag analysis excluding events occurring within the first 2 years of follow-up: keeping inclusion/exclusion criteria and outcome definitions unchanged, participants who experienced the relevant outcome between the baseline date and 2 years of follow-up were treated as early events and excluded, and the primary model was refitted in the remaining sample to evaluate whether the AL–outcome association remained consistent. Second, we addressed missing covariate data using multiple imputation and repeated the primary analyses in the imputed datasets. Two-sided p-values < 0.05 were considered statistically significant. All analyses were conducted in R, version 4.4.1.

## 3 Results

### 3.1 Baseline characteristics

A total of 186,310 participants were included in the incidence analyses (51.84% women; mean age 56.38 years [SD 8.08]). The median follow-up time was 15.39 years (interquartile range [IQR], 14.73–16.01). Baseline characteristics differed markedly across quartiles of AL (Table 1; most comparisons P < 0.0001). Participants in the highest AL quartile (Q4; n = 42,959) were on average older than those in the lowest quartile (Q1; n = 52,726) (58.37 ± 7.43 vs 53.27 ± 8.09 years) and lived in more deprived areas (IMD 17.86 ± 14.03 vs 15.40 ± 12.28). The sex distribution shifted substantially across AL strata, with the proportion of women decreasing from 71.07% in Q1 to 31.80% in Q4 (correspondingly, men increased from 28.93% to 68.20%). The proportion of participants of non-White ethnicity was slightly higher in Q4 than Q1 (5.22% vs 4.10%). Health-related behaviours also varied by AL: smoking (previous/current) increased from 39.70% in Q1 to 52.98% in Q4, whereas alcohol use showed a modest decline across quartiles (55.94% in Q1 to 50.04% in Q4). Importantly, the baseline prevalence of IMIDs rose with increasing AL (5.00% in Q1 to 8.65% in Q4), with broadly similar gradients observed for several individual conditions including rheumatoid arthritis, spondyloarthritis, asthma, inflammatory bowel disease, type 1 diabetes, psoriasis, uveitis, and autoimmune retinopathy; in contrast, systemic lupus erythematosus and myasthenia gravis were rare and did not show a clear monotonic pattern across AL quartiles.

**Table 1:** Baseline characteristics of participants by quartiles of AL.

Variable	Total (n = 186310)	Q1 (n = 52726)	Q2 (n = 64451)	Q3 (n = 26174)	Q4 (n = 42959)	P
Age, year	56.38 ± 8.08	53.27 ± 8.09	56.90 ± 7.93	58.13 ± 7.61	58.37 ± 7.43	<0.0001
IMD	16.40 ± 13.10	15.40 ± 12.28	16.10 ± 12.90	16.75 ± 13.37	17.86 ± 14.03	<0.0001
Sex						<0.0001
Female	96588(51.84)	37475(71.07)	34442(53.44)	11008(42.06)	13663(31.80)	
Male	89722(48.16)	15251(28.93)	30009(46.56)	15166(57.94)	29296(68.20)	
Ethnicity						<0.0001
White	177368(95.20)	50566(95.90)	61299(95.11)	24788(94.70)	40715(94.78)	
Non-white	8942(4.80)	2160(4.10)	3152(4.89)	1386(5.30)	2244(5.22)	
Smoking						<0.0001
Yes	84601(45.41)	20930(39.70)	28476(44.18)	12434(47.51)	22761(52.98)	
No	101709(54.59)	31796(60.30)	35975(55.82)	13740(52.49)	20198(47.02)	
Alcohol use						<0.0001
Higher risk	98143(52.68)	29497(55.94)	33916(52.62)	13235(50.57)	21495(50.04)	
Lower risk	88167(47.32)	23229(44.06)	30535(47.38)	12939(49.43)	21464(49.96)	
IMID						<0.0001
No	173680(93.22)	50088(95.00)	60181(93.37)	24166(92.33)	39245(91.35)	
Yes	12630(6.78)	2638(5.00)	4270(6.63)	2008(7.67)	3714(8.65)	
RA						<0.0001
No	183776(98.64)	52206(99.01)	63581(98.65)	25751(98.38)	42238(98.32)	
Yes	2534(1.36)	520(0.99)	870(1.35)	423(1.62)	721(1.68)	
SpA						<0.0001
No	185640(99.64)	52623(99.80)	64245(99.68)	26056(99.55)	42716(99.43)	
Yes	670(0.36)	103(0.20)	206(0.32)	118(0.45)	243(0.57)	
Asthma						<0.0001
No	180618(96.94)	51409(97.50)	62538(97.03)	25274(96.56)	41397(96.36)	
Yes	5692(3.06)	1317(2.50)	1913(2.97)	900(3.44)	1562(3.64)	
IBD						<0.0001
No	184766(99.17)	52372(99.33)	63893(99.13)	25937(99.09)	42564(99.08)	
Yes	1544(0.83)	354(0.67)	558(0.87)	237(0.91)	395(0.92)	
SLE						0.03
No	186150(99.91)	52683(99.92)	64383(99.89)	26148(99.90)	42936(99.95)	
Yes	160(0.09)	43(0.08)	68(0.11)	26(0.10)	23(0.05)	
TIDM						<0.0001
No	185374(99.50)	52655(99.87)	64143(99.52)	26023(99.42)	42553(99.05)	
Yes	936(0.50)	71(0.13)	308(0.48)	151(0.58)	406(0.95)	
MG						0.06
No	186211(99.95)	52710(99.97)	64409(99.93)	26158(99.94)	42934(99.94)	
Yes	99(0.05)	16(0.03)	42(0.07)	16(0.06)	25(0.06)	
Psoriasis						<0.0001
No	184481(99.02)	52387(99.36)	63880(99.11)	25890(98.91)	42324(98.52)	
Yes	1829(0.98)	339(0.64)	571(0.89)	284(1.09)	635(1.48)	
Uveitis						0.05
No	186041(99.86)	52669(99.89)	64356(99.85)	26129(99.83)	42887(99.83)	
Yes	269(0.14)	57(0.11)	95(0.15)	45(0.17)	72(0.17)	
AIR						<0.0001
No	186093(99.88)	52688(99.93)	64387(99.90)	26139(99.87)	42879(99.81)	
Yes	217(0.12)	38(0.07)	64(0.10)	35(0.13)	80(0.19)	

Participant characteristics were summarized using means ± standard deviations for continuous variables and frequencies (percentages) for categorical variables. IMD: index of multiple deprivation, AL: allostatic load, IMID: immune-mediated inflammatory disease, CI: confidence interval, RA: rheumatoid arthritis, SpA: spondyloarthritis, IBD: inflammatory bowel disease, T1DM: type 1 diabetes mellitus, MG: Myasthenia Gravis, AIR: autoimmune retinopathy, SLE: Systemic lupus erythematosus.

### 3.2 Cox regression results

Overall, higher AL levels—whether analysed as a continuous score or in quartiles—were positively associated with the incidence of RA, SpA, asthma, T1DM, psoriasis, IBD, AIR and “any IMID” (Table 2). Treated as a continuous variable, every one-point increase in AL was linked to an 8 % rise in the risk of developing any IMID (HR = 1.08, 95 % CI 1.07–1.09), with T1DM showing the steepest dose–response gradient (HR = 1.24, 95 % CI 1.20–1.28). In the quartile model, participants in the second, third and fourth AL quartiles (Q2–Q4) all exhibited significantly higher IBD risk than those in the lowest quartile (Q1). Notably, T1DM displayed an especially strong association in Q4 (HR = 5.16, 95 % CI 3.98–6.71). By contrast, SLE, MG and uveitis showed no clear relation to AL. For more detailed information, please refer to Table S4.

**Table 2:** Cox proportional-hazards model results for the association between allostatic load quartiles and the incidence risk of IMIDs.

Disease	Q1	Q2 β (95% CI)	Q3 β (95% CI)	Q4 β (95% CI)	*p for trend*
All IMIDs	Ref	1.22(1.16, 1.29)	1.39(1.30, 1.47)	1.55(1.47, 1.63)	
*p*		<0.001	<0.001	<0.001	<0.001
RA	Ref	1.21(1.09, 1.36)	1.44(1.26, 1.64)	1.52(1.35, 1.72)	
*p*		<0.001	<0.001	<0.001	<0.001
SpA	Ref	1.53(1.20, 1.95)	2.08(1.58, 2.73)	2.50(1.95, 3.19)	
*p*		<0.001	<0.001	<0.001	<0.001
Asthma	Ref	1.13(1.06, 1.22)	1.31(1.20, 1.43)	1.38(1.28, 1.50)	
*p*		<0.001	<0.001	<0.001	<0.001
IBD	Ref	1.21(1.05, 1.38)	1.22(1.03, 1.44)	1.19(1.02, 1.38)	
*p*		0.007	0.025	0.029	0.067
SLE	Ref	1.36(0.92, 2.01)	1.38(0.84, 2.29)	0.83(0.49, 1.41)	
*p*		0.13	0.2	0.5	0.591
T1DM	Ref	3.00(2.31, 3.89)	3.33(2.50, 4.44)	5.16(3.98, 6.71)	
*p*		<0.001	<0.001	<0.001	<0.001
MG	Ref	1.56(0.87, 2.79)	1.28(0.63, 2.61)	1.15(0.60, 2.20)	
*p*		0.14	0.5	0.7	0.873
Psoriasis	Ref	1.24(1.08, 1.42)	1.44(1.23, 1.70)	1.87(1.62, 2.15)	
*p*		0.002	<0.001	<0.001	<0.001
Uveitis	Ref	1.17(0.84, 1.64)	1.29(0.86, 1.93)	1.24(0.85, 1.79)	
*p*		0.4	0.2	0.3	0.267
AIR	Ref	1.05(0.70, 1.58)	1.30(0.81, 2.08)	1.77(1.18, 2.66)	
*p*		0.8	0.3	0.006	0.001

IMID: immune-mediated inflammatory disease, CI: confidence interval, RA: rheumatoid arthritis, SpA: spondyloarthritis, IBD: inflammatory bowel disease, T1DM: type 1 diabetes mellitus, MG: Myasthenia Gravis, AIR: autoimmune retinopathy, SLE: Systemic lupus erythematosus.

Among individuals who already had an IMID, RA, asthma, IBD or T1DM at baseline, higher AL was significantly linked to increased all-cause mortality, with an evident dose–effect pattern (Table S5). For any IMID, each one-point rise in AL corresponded to an HR of 1.11 (95 % CI 1.09–1.13), and the top AL quartile carried an HR of 2.01 (95 % CI 1.75–2.30). Among RA patients, the Q4 HR reached 6.59 (95 % CI 2.63–16.5). Associations for SpA, SLE, myasthenia gravis, psoriasis, uveitis and AIR did not attain statistical significance.

### 3.3 Interaction analysis

#### 3.3.1 Multiplicative interaction

Multiplicative-scale analyses indicated heterogeneous synergistic effects of AL with several modifiers (Table S7-S8). For RA incidence, significant interactions were observed between AL and physical activity (HR = 0.95, 95 % CI 0.91–1.00) and between AL and moderate PRS (HR = 0.93, 95 % CI 0.88–0.98; p = 0.008). For asthma, AL interacted significantly with sleep score (HR = 1.01, 95 % CI 1.00–1.03) and with both moderate and high PRS (moderate PRS: HR = 0.97, 95 % CI 0.93–1.00; high PRS: HR = 0.96, 95 % CI 0.92–1.00). Significant interactions were also found between AL and sleep score for IBD risk (HR = 1.03, 95 % CI 1.00–1.05) and between AL and high PRS for T1DM risk (HR = 0.88, 95 % CI 0.80–0.97).

#### 3.3.2 Additive interaction

In this study, we found that several factors had statistically significant additive interactions with allostatic load; notably, some protective factors mitigated the allostatic-load–driven increase in the incidence of IMIDs (Table S9 and S10). For RA, AL and physical activity displayed a negative interaction (attributable proportion due to interaction [AP] = −4.98%, 95% CI − 8.01% to −1.95%). T1DM exhibited multiple interactions: AL × physical activity (AP = −6.50%, 95% CI − 11.3% to −1.68%); AL × ω-3 PUFA (AP = −9.74%, 95% CI − 14.2% to −5.24%); and, for the ω-3 subtype docosahexaenoic acid (DHA), AL × DHA (AP = −7.94 %, 95% CI − 11.7% to −4.19%). In prognostic analyses, AL interacted additively with ω-3 PUFA for all-cause mortality in T1DM (AP = −4.65%, 95% CI − 10.6% to −1.29%) and with physical activity for mortality in asthma (AP = −4.99%, 95 % CI − 8.41 % to −1.57%).

Conversely, AL showed positive synergistic effects with high PRS, increasing the risk of SpA (AP = 7.60 %, 95 % CI 2.46 %–12.7 %), T1DM (AP = 4.71%, 95% CI 2.36%–7.05%) and psoriasis (AP = 4.91%, 95 % CI 2.25%–7.57%).

### 3.4 Sensitivity analyses

Sensitivity checks—excluding events occurring within the first two years of follow-up and repeating the analyses after multiple imputation for missing covariate data—did not materially alter the magnitude or significance of the associations between AL and each IMID, confirming the robustness of the primary findings (Table S11 and S12).

## 4 Discussion

Drawing on a large, population-based cohort, our study delineates the causal landscape linking AL to the onset and progression of IMIDs. We observed clear dose–response relations whereby higher AL levels predicted greater incidence of RA, SpA, asthma, T1DM, psoriasis and AIR. Accumulated AL was likewise associated with markedly elevated all-cause mortality among patients with RA, asthma, IBD and T1DM. Additive-scale interaction analyses further showed that physical activity and ω-3 PUFAs antagonised AL-related IMID risk in selected outcomes, whereas high genetic susceptibility synergised with AL exposure in a disease-specific manner—underscoring the multilayered interplay among genetic vulnerability, environmental stressors and immune regulation.

Our study demonstrated a robust association between AL and prototypical rheumatic IMIDs such as RA and SpA, corroborating an existing body of evidence. For example, 22 years of follow-up in the Nurses’ Health Study II (NHS-II) showed that post-traumatic stress disorder was linked to a markedly higher risk of incident RA (HR = 1.76, 95 % CI 1.05–2.43), an association that remained stable after adjustment for smoking history and multiple sociodemographic factors [18]. Likewise, a French longitudinal cohort observed that sudden stressful life events raised the Bath Ankylosing Spondylitis Disease Activity Index (BASDAI), supporting a temporal relation between stress and SpA-related inflammation [19]. Clinically, we found that cumulative AL exacerbated mortality risk among RA patients, echoing the meta-analysis by Haley and colleagues, in which a high AL burden increased all-cause mortality by ≈22 % (95 % CI 1.11–1.41) and cardiovascular mortality by ≈31 % (95 % CI 1.10–1.57) [20]. Sophia et al. further confirmed that the excess mortality in RA is driven primarily by cardiovascular events and malignancies [21].

Regarding mechanisms, because this is a population-based cohort study, our findings primarily support causal inference rather than direct mechanistic validation. We therefore present only an interpretive framework that is consistent with our observations and supported by prior evidence. On the one hand, the chronic stress state captured by AL may, through sustained dysregulation of the sympathetic nervous system and the hypothalamic–pituitary–adrenal axis, passively “reset” immune homeostasis, rendering inflammatory responses more readily triggered and less likely to be terminated [4,22]. In parallel, prolonged stress may disrupt glucocorticoid rhythmicity and reduce receptor sensitivity, thereby constraining their anti-inflammatory and immunosuppressive effects and fostering a physiological milieu prone to persistent, low-grade inflammation [23]. This framework offers a coherent explanation for the elevated risks of multisystem IMIDs observed in our analyses; however, the specific molecular steps will require confirmation through experimental studies and longitudinal investigations incorporating repeated biomarker assessments.

From a cross-organ clinical perspective, we observed that higher AL was closely associated with both incident disease and adverse outcomes in T1DM, psoriasis, asthma, and IBD, suggesting that chronic stress burden may act as a shared pro-inflammatory “background noise” across tissue-specific immune responses. This pattern is broadly consistent with prior evidence.

For example, the prospective All Babies in Southeast Sweden (ABIS) cohort reported that severe early-life events were associated with a threefold higher risk of childhood T1DM (HR = 3.0, 95% CI: 1.6–5.6) [24], potentially via cytokine-driven insulitis stress-hormone–related metabolic effects [25]. For psoriasis, a systematic review of 39 studies found that 46% of patients reported a temporal link between psychological stress and symptom exacerbation, implicating stress-related neuro–immune modulation in amplifying cutaneous inflammation beyond immune imbalance alone [22,26]. In addition, our findings linking AL to the risk and prognosis of asthma and IBD align with prior studies and patient-reported evidence [27,28], supporting a clinically plausible model in which dysregulated bidirectional brain–immune signaling may exert cross-organ effects in the airways and gut.

Of particular interest, our study suggests a putative causal link between AL and AIR. Direct evidence for stress–AIR associations remains limited, but supporting clues exist. In a cross-sectional study, Elyse et al. found that higher scores on the Perceived Stress Scale-10 were significantly associated with non-infectious uveitis (coefficient = 4.3, 95 % CI 1.8–6.9) [29]. Experimental data by Wang et al. show that chronic social defeat stress irreversibly impairs retinal arteriolar autoregulation and endothelial function in mice [30], while Leoné et al. reported that stress exposure heightens the risk of retinal ischaemia and astrocyte damage [31]. Collectively, these findings imply that chronic stress may undermine the eye’s immune-privileged status through pro-inflammatory effects and blood–retina-barrier disruption.

Regarding modifiable factors, our additive interaction analyses suggest that healthier lifestyles may partially offset AL-related risk. For instance, ω-3 PUFA showed a more-than-additive protective association with T1DM among individuals with high AL (total ω-3 PUFA AP = −9.7%; DHA AP = −7.9%), consistent in direction with evidence that infant cod-liver-oil supplementation is linked to a lower risk of T1DM [32]. Plausibly, this relates to the anti-inflammatory properties of ω-3 PUFA and their modulation of neuroendocrine stress responses [33]. Similarly, we observed protective associations of regular physical activity with incident RA and asthma progression, in line with prior epidemiologic findings [34,35], potentially reflecting combined effects on lowering inflammatory burden and improving cardiorespiratory fitness [[35], [36], [37]].

At the genetic level, we observed disease-heterogeneous positive interactions between higher genetic susceptibility and AL exposure for several IMIDs—most notably type 1 diabetes, spondyloarthritis, and psoriasis—suggesting that genetic burden may amplify the impact of chronic stress load on immune dysregulation. One plausible explanation is that sustained stress may trigger inflammatory cascades through epigenetic reprogramming [38], underscoring the nuanced synergy between inherited liability and environmental stressors in IMIDs.

Limitations should also be acknowledged. First, AIR is diagnostically challenging: multimodal data integration (e.g. electroretinography, optical coherence tomography and genomics) is still evolving, and ocular-specific autoimmune signals are difficult to capture in peripheral blood [39]. A validated polygenic-risk-score framework for AIR is therefore lacking, creating data gaps. Second, UK Biobank does not distinguish infectious from non-infectious uveitis, and this taxonomic imprecision may introduce information or confounding bias, diluting AL–uveitis associations. Third, although our biomarker panel followed commonly used AL constructs and reflected UKB variable availability [8], those constraints may limit cross-cohort comparability. Finally, the cohort is predominantly middle-aged and older White adults; early-onset IMID trajectories may be under-represented, and caution is needed when extrapolating our results to non-European populations.

## 5 Conclusion

In summary, this large-scale cohort study demonstrates a clear dose-response, causal relationship between AL—a biomarker of cumulative chronic stress—and both the incidence and prognosis of multiple IMIDs. Our findings suggest that healthy lifestyle factors, such as regular physical activity and higher intake of ω-3 PUFAs, can buffer part of the pathogenic impact of elevated allostatic load, whereas high genetic susceptibility amplifies it for certain IMIDs. From an integrated psychoneuroimmunology perspective, the interplay among environmental stress, lifestyle modification and genetic background offers a new biomarker framework and evidence base for IMID risk prediction and stratified intervention strategies.

## CRediT authorship contribution statement

Ziling Yang: Conceptualization, Methodology, Formal analysis, Data curation, Visualization, Writing–original draft. Jinming Zhang: Conceptualization, Methodology, Formal analysis, Data curation, Visualization, Writing–original draft. Zhong Qu: Conceptualization, Methodology, Formal analysis, Data curation, Visualization. Zhuo Zhao: Conceptualization, Methodology, Validation, Writing–review & editing, Funding acquisition. Yajuan Zheng: Conceptualization, Methodology, Validation, Writing–review & editing.

## Declaration of Generative AI and AI-assisted technologies in the writing process

The authors confirm that no generative AI or AI-assisted technologies were used in the writing of this manuscript, nor in the preparation of figures, images, or artwork.

## Funding

This study was financially supported by the Clinical Research Management Committee of the First Hospital of Jilin University (Approval No.2024-HS-174).

## Data availability

Data may be obtained from a third party and are not publicly available. The UK Biobank data are available on application at https://www.ukbiobank.ac.uk.

## Declaration of competing interest

The authors declare that they have no known competing financial interests or personal relationships that could have appeared to influence the work reported in this paper.
