# Supplementary material for: Allostatic load elevates the risk and adverse prognosis of immune-mediated inflammatory diseases: modulatory effects of lifestyle interventions and genetic susceptibility
Source: J Nutr Health Aging. 2026 Jan 24;30(3):100792. doi: 10.1016/j.jnha.2026.100792 (PMC12860939; doi:10.1016/j.jnha.2026.100792)
Supplement: Supplementary file 1 [file mmc1.docx]

| **Table S1. Definitions of allostatic load.** | |
| --- | --- |
| **Biomarkers** | |
| metabolic system | glycated hemoglobin (HbA1c) |
|  | triglycerides (TG)，mmol/L |
|  | high-density lipoprotein cholesterol (HDL)，mmol/L |
|  | low-density lipoprotein cholesterol (LDL)，mmol/L |
|  | total cholesterol (TC)，mmol/L |
|  | waist-to-hip ratio (WHR) |
| cardiovascular system | systolic blood pressure (SBP) |
|  | diastolic blood pressure (HBP) |
|  | pulse rate (PR) |
| inflammatory or immune system | C-reactive protein (CRP), mg/L |
|  | insulin-like growth factor-1 (IGF-1) |
| kidney function | creatinine (Cre) |
| **Method of calculation** | |
| Patients with HbA1c, LDL, TC, TG, WHR, SBP, DBP, PR, CRP, and Cre in the 75th percentile or higher were awarded a point. Conversely, HDL and IGF-1 in the 25th percentile or lower was awarded a point. An addition of the scores from all selected factors was used to calculate the allostatic load (AL) score, which ranged from 0 to 12. We employed 3 classification methods to capture the effect size of AL on observed outcomes. For classification 1 (AL-median groups), we dichotomized into high and low categories, where high AL was defined as scores exceeding the median. We also grouped the composite AL summary score into quartiles. Moreover, to capture the dose-response relationship, we initially treated the AL score as a continuous variable(1). | |

| **Table S2. ICD-10 codes of immunologically mediated inflammatory diseases.** | |
| --- | --- |
| Disease | ICD-10 code |
| RA | "M050","M0500","M0504","M0509","M051","M0510","M0517","M0518","M0519","M052","M0520","M0696","M0524","M0526","M0528","M0529","M053","M0530","M0538","M0539","M058","M0580","M0582","M0639","M0583","M0584","M0586","M0587","M0588","M0589","M059","M0590","M0591","M0592","M0593","M0638","M0594","M0595","M0596","M0597","M0598","M0599","M0600","M0601","M0602","M0603","M0636","M0637","M0604","M0605","M0606","M0607","M0608","M0609","M060","M0621","M0622","M0625","M0626","M0627","M0699","M061","M0610","M0611","M0616","M0619","M062","M0620","M063","M0630","M0632","M0633","M0634","M0698","M064","M0640","M0641","M0643","M0644","M0645","M0646","M0647","M0649","M068","M0680","M0681","M0697","M0682","M0684","M0685","M0686","M0687","M0689","M0688","M069","M0690","M0691","M0692","M0693","M0694","M0695" |
| SpA | "M45X0", "M45X1", "M45X2", "M45X3", "M45X4", "M45X5", "M45X6", "M45X7", "M45X8","M45X9","M070","M0700","M0701","M0703","M0704","M0707","M0709","M071","M0717","M0719","M072","M0720","M0726","M0728","M0729","M073","M0730","M0731","M0732","M0733","M0734","M0735","M0736","M0737","M0738","M0739","M023","M0230","M0233","M0236","M0238","M0239","M028","M0280","M0281","M0284","M0285","M0286","M0287","M029","M0290","M0293","M0294","M0295","M0296","M0297","M0299","M031","M0320","M0326","M0360","M0369","M074","M0740","M0741","M0746","M0747","M0749","M075","M0750","M0751","M0755","M0758","M0759","M076","M0760","M0764","M0766","M0769", 'M468','M4680','M4682','M4684','M4686','M4687','M4688', 'M4689', 'M0810', 'M0819' |
| IBD | "K500", "K501","K508", "K509","K510", "K511","K512", "K513","K514", "K515", "K518","K519" |
| Asthma | "J450", "J451", "J458", "J459" |
| T1DM | "E100","E101","E102","E108","E109","E103","E104","E105","E106","E107" |
| MG | "G700" |
| Psoriasis | "L400","L401","L403","L408","L409","L404","L405" |
| Uveitis | "H200", "H201","H202","H208","H209","H220" |
| SLE | "M320", "M321","M328","M329","M3290" |
| AIR | "H350" |

RA: rheumatoid arthritis, SpA: spondyloarthritis, IBD: inflammatory bowel disease, T1DM: type 1 diabetes mellitus, MG: Myasthenia Gravis, AIR: autoimmune retinopathy, SLE: Systemic lupus erythematosus.

| **Table S3. Definitions for covariates and interaction variables.** | |
| --- | --- |
| **Covariates** | **UK Biobank** |
| Age at recruitment | This is a derived variable based on the date of birth and date of attending an initial assessment center and refers to the age of the participant on the day they attended an Initial Assessment Centre, truncated to the whole year. (variable handling: as a continuous variable) |
| Sex | A mixture of the sex the National Health Service had recorded for the participant and self-reported sex. (variable handling: categorical variable “Female”, “Male”) |
| Ethnicity | Self-reported: “What is your ethnic background”. We classified the variable into: White (White) and Non-white (Mixed, Asian or Asian British, Black or Black British, Chinese, and other ethnic groups) because the number of non-white ethnic backgrounds was too small |
| Index of Multiple Deprivation (IMD) | The higher, the more socioeconomic deprivation one was suffering. IMD was a measure of relative deprivation for small areas (Lower Layer Super Output Areas, LSOAs) used to rank neighborhoods across the UK according the output area in which their postcode was located. IMD was generated based on 37 seven separate indicators, and was composed of seven domains of deprivation according to their respective weights, including Income score (26411, 22.5 %), Employment score (26412, 22.5 %), Health score (26413, 13.5 %), Education score (26414, 13.5 %), Housing score (26415, 9.3 %), Crime score (26416, 9.3 %) and Living environment score (26417, 9.3 %). Each IMD domain is ranked from the least to most deprived. (variable handling: as continues variable) |
| Smoking | Self-reported current/past smoking status of the participant. We classified the variable handlings into 0 (Never) and 1 (Previous, current). |
| Alcohol use | Participants self-reported the number of alcohol units (10 ml of pure ethanol) consumed, in “units per week” (for frequent drinkers) or “units per month” (for less frequent drinkers), across several beverage categories (red wine, white wine/champagne, beer/cider, spirits, fortified wine, and “other”). We classified the variable into: 1 (daily consumption of one drink or fewer for women and two drinks or fewer for men, according to the dietary guidelines in the UK) and 0 (consumption beyond these limits). |
| **Interaction variables** |  |
| Sleep score(2) | Self-reported UK Biobank touchscreen sleep questionnaire. We defined the variable reflecting a composite sleep quality score according to previous studies using UK Biobank data. This included the following five components: 1. Sleep duration: 7-8 hours/day 2. Chronotype: Early or early-to-bed/early-to-rise 3. Insomnia: Rarely or never 4. Snoring: None 5. Daytime sleepiness: Rarely or never We classified the variable handlings into 1 (low-risk sleep behavior for all 5 components) and 0 (high-risk sleep behavior in at least one component). Higher scores indicated better sleep quality. |
| Physical activity | Self-reported: UK Biobank physical activity questionnaire (IPAQ short form). We classified the variable handlings into 1 (150 minutes moderate activity per week OR ≥ 75 minutes vigorous activity per week OR equivalent combination OR moderate physical activity at least 5 days a week or vigorous activity once a week) and 0 (below adequate level) recommended by the American Heart Association. |
| Noise exposure(3) | Noise exposure was assessed using the “average 24-h sound level of noise pollution” metric, which provides a comprehensive measure of daily noise levels. This metric primarily reflects the general ambient noise levels in the living environments of the study participants. To maintain consistent scaling and enhance interpretability, the original noise level values were divided by 10 in the calculations. |
| Green space and water space | The percentage of the total proportion of all land-use categories based on the 2005 Generalized Land Use Database (GLUD) for England (Department for Communities and Local Government, 2007) was used to estimate the percentage of residential green space and blue space, which were classified as “greenspace” and “water.” To assess the interaction of GBN density with AL, the ratio of green and blue space within the 300 m buffer was considered. To maintain consistent scaling and enhance interpretability, the original greenspace and water level values were divided by 10 in the calculations. |
| Combined air pollution exposure(4) | The land use regression (LUR)-based model was used to estimate the annual average concentration of particulate matter of fine inhalable particles, with diameters that are generally 2.5 μm and smaller (PM2.5), particulate matter of inhalable particles, with diameters that are generally 10 μm and smaller (PM10), nitrogen dioxide (NO2), and nitrogen oxides (NOx), as previously described. The combined air pollution exposure score was estimated as follows. Firstly, a multivariate Cox regression was applied to obtain the beta value of each pollutant, then the total effect of air pollution was developed by using the formula [(βPM2.5 ∗ PM2.5 + βPM10 ∗ PM10 + βNO2 ∗ NO2 + βNOx ∗ NOx) ∗ (4/sum of the β coefficients)]. In calculation of each β value, taking βPM2.5 as an example, we first included participants' exposure to PM2.5 into the multivariable Cox proportional hazards model to estimate its effect on the risk of IMID, and the partial regression coefficients for PM2.5 is βPM2.5. |
| Omega-3(5) | The plasma fatty acids level in UK Biobank were quantified using targeted high-throughput nuclear magnetic resonance metabolomics, conducted by Nightingale Health on non-fasting EDTA plasma samples. Omega-3 PUFAs and DHA results were used in this study. Calculations were performed using tertiles of the original values, ensuring a balanced distribution across exposure levels while preserving interpretability and statistical robustness. |
| PRS | We constructed the polygenic risk score (PRS) using the clumping and threshold (C-T) method. Specifically, we drew upon relevant GWAS findings for each disease of interest, identifying the most significant single nucleotide polymorphisms (SNPs) within a 1000-kb window, at an r^^2^ threshold above 0.1, and retaining those with p≤5×10^^-8^ for the final linkage disequilibrium (LD) cluster. In this study, the PRS is categorized into three genetic risk strata: low (lowest quintile), moderate (quintiles 2–4), and high (highest quintile). The PubMed identifiers (PMIDs) for each disease-specific GWAS source used in PRS computation are provided below. Rheumatoid arthritis: 36333501 Spondyloarthritis(SpA): 23749187 Asthma: 29273806 Inflammatory bowel disease(IBD): 26192919 Type 1 diabetes: 25751624 Psoriasis: 23143594 Autoimmune retinopathy: 26502338 |

**Reference:**

1. Zhao J, Xue E, Zhou S, Zhang M, Sun J, Tan Y, et al. Allostatic load, genetic susceptibility, incidence risk, and all-cause mortality of colorectal cancer. J Natl Cancer Inst. 2025;117(1):134-43.

2. Zhou Q, Liu S, Chen J, Tuersun Y, Liang Z, Wang C, et al. The role of sleep quality and anxiety symptoms in the association between childhood trauma and self-harm attempt: A chain-mediated analysis in the UK Biobank. J Affect Disord. 2024;362:569-77.

3. Yang T, Hu X, Wang J, Rao S, Cai YS, Li G, et al. Long-Term Exposure to Road Traffic Noise and Incident Heart Failure: Evidence From UK Biobank. JACC Heart Fail. 2023;11(8 Pt 1):986-96.

4. Chen J, Zhang H, Fu T, Zhao J, Nowak JK, Kalla R, et al. Exposure to air pollution increases susceptibility to ulcerative colitis through epigenetic alterations in CXCR2 and MHC class III region. EBioMedicine. 2024;110:105443.

5. Xue CC, Li H, Yu M, Chong CCY, Fan Q, Tham YC, et al. Omega-3 Fatty Acids as Protective Factors for Age-Related Macular Degeneration: Prospective Cohort and Mendelian Randomization Analyses. Ophthalmology. 2025;132(5):598-609.
